# Iron deficiency across chronic kidney disease stages: Is there a reverse gender pattern?

**DOI:** 10.1371/journal.pone.0191541

**Published:** 2018-01-22

**Authors:** Mabel Aoun, Rita Karam, Ghassan Sleilaty, Leony Antoun, Walid Ammar

**Affiliations:** 1 Department of Nephrology, Saint-Joseph University, Beirut, Lebanon; 2 Ministry of Public Health, Beirut, Lebanon; 3 Faculty of sciences and medical sciences, Lebanese University, Beirut, Lebanon; 4 Department of Epidemiology and Biostatistics, School of medicine, Saint-Joseph University, Beirut, Lebanon; 5 Department of internal medicine, Holy Spirit University, Kaslik, Lebanon; Universidade Estadual Paulista Julio de Mesquita Filho, BRAZIL

## Abstract

In non-dialysis chronic kidney disease patients, looking for iron deficiency is highly variable in practice and there is a great variability regarding the cutoffs used to treat iron deficiency. The aim of this study is to investigate the degree of iron deficiency in non-dialysis chronic kidney disease patients on erythropoiesis-stimulating agents. We included all non-dialysis chronic kidney disease patients that applied to the Lebanese Ministry of Public Health for erythropoiesis-stimulating agents’ coverage during a 5-month period. Iron requirement was assessed based on two guidelines’ target-to-treat cutoffs: 1-ferritin <100 ng/ml and/or TSAT < 20% (KDOQI 2006), 2- ferritin ≤500 ng/ml and TSAT ≤30% (KDIGO 2012). A total of 238 CKD patients were included over 5 months. All patients had a ferritin level in their record and 64% had an available TSAT. Median age was 71.0 (59.8–79.3) years and 61.8% were female. All had an eGFR<60 ml/min. The proportion of patients found to require iron therapy ranged between 48 and 78% with a trend towards higher values when using KDIGO-based criteria. Using ANCOVA test, inverse normal transformations of ferritin and TSAT showed a reverse pattern between men and women with women being more iron deficient in the early stage. Iron deficiency is highly prevalent in non-dialysis chronic kidney disease patients on erythropoiesis-stimulating agents’ therapy. These findings reflect a lack in effective iron supplementation when managing anemia in pre-dialysis patients, especially in men at advanced stages. Renal societies should spread awareness about iron deficiency screening in those patients.

## Introduction

Anemia prevalence worldwide was estimated at 33% in 2010 with iron deficiency being the leading cause in half of the cases [1,2]. In chronic kidney disease (CKD) patients, anemia is a clinically significant burden and it becomes more prevalent with declining glomerular filtration rate (GFR) [3]. Anemia is associated with reduced quality of life and increased cardiovascular morbidity and mortality [4]. Erythropoietin (EPO) deficiency remains the major cause of anemia in CKD patients due to the decrease in renal EPO production [5]. However, after the release on the market of the recombinant human EPO (r-HuEPO) in the 1990s and the decline in blood transfusions, iron deficiency started to emerge as an important cause of anemia in CKD patients.

Iron is essential for oxygen binding in red blood cells and is crucial for many other cellular functions. Iron deficiency, even without anemia, has been demonstrated to be an independent risk factor for increased mortality [6]. Nephrologists have been focusing on iron deficiency in hemodialysis patients and intravenous iron is routinely administered during the dialysis sessions. Iron loss in dialysis patients has been related to several factors such as blood loss from dialyzer and tubing, regular blood tests, impaired dietary iron absorption and gastrointestinal losses [7]. On the other hand, in non-dialysis CKD (ND-CKD) patients, looking for iron deficiency is highly variable in practice and using intravenous iron as a treatment at this stage is less common. The major cause of iron deficiency in ND-CKD patients is believed to be a low diet intake in addition to the decrease in iron absorption due to the high levels of hepcidin [8]. The depletion of circulating iron can also result from the enhanced erythropoiesis with erythropoiesis-stimulating agents (ESAs) [8].

Several studies have addressed worldwide the prevalence of iron deficiency in ND-CKD patients and there is a great variability regarding the cut-offs used to define iron deficiency. In addition, little is known about iron deficiency in Lebanese ND-CKD patients. Lebanon is an Eastern Mediterranean developing country where almost half of the population benefits from health coverage of the Lebanese Ministry of Public Health (MOPH). In order to receive ESA therapy, Lebanese CKD patients apply to the MOPH using a requisition form prefilled by their treating nephrologist and a recent blood test including serum hemoglobin, creatinine, ferritin and if available transferrin saturation (TSAT). ESA therapy approval is then renewed every three to six months. In this study, we reviewed all applications to the MOPH for maintenance ESA therapy over 5 months in order to investigate the degree of iron deficiency in ND-CKD patients on ESA therapy.

## Materials and methods

### Study design and participants

This is a national observational study that included all ND-CKD patients that applied to the Lebanese MOPH for ESA treatment during a 5-month period from July 2016 till end of November 2016.

Patients <18 years old, kidney transplant patients and patients with serum ferritin missing values were excluded. Duplicate names were excluded.

Data were collected on the following parameters: demographic factors (age, gender), hemoglobin (Hb) level, serum creatinine (mg/dl), serum ferritin (ng/ml) and serum transferrin saturation (TSAT) if available.

The study was approved by the ethics committee of the Saint-Joseph University-Beirut (approval number CEHDF 983). It complies with the Declaration of Helsinki of 1975. The ethics committee waived the need for consent of patients and data were collected anonymously.

### Definitions

GFR was estimated for each patient using the CKD-EPI equation. CKD stages were defined based on the Kidney Disease: Improving Global Outcomes (KDIGO) 2012 Clinical Practice classification [9]: stage 3, eGFR 30–59 ml/min; stage 4, eGFR 15–29 ml/min and stage 5, eGFR < 15 ml/min. In this study, stage 3 will be referred to as early stage of CKD and stages 4 and 5 as advanced stages.

Severe anemia was defined as Hb <10 g/dl and patients were assessed based on two groups: those with Hb<10 g/dl and those with Hb <11 g/dl. The rationale behind this is the fact that KDIGO guidelines recommend starting ESA therapy in ND-CKD patients when Hb falls to less than 10 g/dl and they set a therapeutic target not exceeding 11.5 g/dl [10].

Iron requirement was assessed by using two definitions: 1-ferritin <100 ng/ml and/or TSAT < 20% (based on the KDOQI target-to-treat cutoffs and NICE guidelines), 2- ferritin ≤500 ng/ml and TSAT ≤30% (based on the KDIGO 2012 target-to-treat cutoffs) [4,10,11].

### Statistical analysis

Shapiro-Wilk and Kolmogorov-Smirnov tests were used to assess significant departure from normality. Age distribution was heavily skewed towards upper values. A power Box-Cox transformation yielded λ = 2 as the optimal parameter to bring the distribution of age towards normality.

TSAT was not available for 36% of the patients. Missing value analysis was undertaken to check the assumption of MCAR (Missing Completely At Random) using Little’s test. For main analysis, the original data were used. For sensitivity analysis, missing values were imputed using multiple imputation algorithms (iterative Markov chain Monte Carlo), generating 5 datasets with imputed values, from which parameters were averaged.

Ferritin, TSAT and serum creatinine were severely skewed to the left. Although power transformation would have potentially corrected for departure from normality, it would have been difficult to interpret the resulting parameters in a clinically meaningful way. Thus, an inverse normal transform (INT) approach was elected, using Van der Warden ranks. Regarding ferritin: a 2-way ANCOVA was utilized using INT of ferritin rank as dependent variable, CKD stage and gender as independent factors, and adjusting for hemoglobin and the Box-Cox transform of age as covariates. The assumptions of the model were assessed (normality of sampling distributions, linearity, homoscedasticity, and homogeneity of regression). A similar approach was used for INT of TSAT rank. Although the Spearman correlation between ferritin levels and TSAT was moderate (rho = 0.45), thus enabling a 2-way MANCOVA model, a 2 separate ANCOVA models’ approach was elected over the former due to missing values in TSAT.

The two definitions of iron deficiency were analyzed in relation to gender and CKD using the Mantel-Haenzel adjusted OR. Since the latter criteria were not available for the whole sample due to missing values in TSAT variable, sensitivity analysis was conducted by imputing derived values from multiple imputation (scenario 1), then imputing either positive (scenario 2) or negative criteria (Scenario 3) for all missing values. Agreement between the 2 criteria systems was calculated for base case and for sensitivity analyses using Cohen’s kappa. When applicable, 95% CIs by bootstrapping were calculated.

## Results

### Patients’ characteristics

A total of 238 CKD subjects were included over 5 months. Their age ranged between 22 and 94 years. Their characteristics are summarized in Table 1. Ferritin assay records were complete. TSAT could not be retrieved for 85 patients (36%). Missing value analysis taking into account information from all the variables in the dataset did not reject the hypothesis of TSAT as being MCAR (Missing Completely At Random), with Little's MCAR p-value = .496. That is, characteristics of patients with missing TSAT information did not statistically differ from those with available TSAT information. Accordingly, missing TSAT information was imputed using a multiple imputation algorithm (Cf. Statistical Methods). Table 1 shows original TSAT statistics (n = 153) and imputed TSAT statistics (n = 238), the latter being the average of 5 generated datasets with imputed values.

**Table 1:** Characteristics of the 238 patients.

Source	Variable	Statistic	Result
**Original**	Age (years)*	Median (IQR)	71.0 (59.8–79.3)
	**eGFR (ml/min**)*	Median (IQR)	16 (11–27)
	**Female/Male**	Ratio	147/91 = 1.62/1
	**CKD stage 3**	N (%)	43 (18.1)
	**CKD stage 4**	N (%)	96 (40.3)
	**CKD stage 5**	N (%)	99 (41.6)
	**Hemoglobin (g/dl)**	Mean ± SD	9.6 ± 1.4
	**Hb < 10 g/dl**	N (%)	135 (56.7%)
	**Hb < 11 g/dl (%)**	N (%)	192 (80.7%)
	Ferritin level (ng/ml)*	Median (IQR)	180.5 (82.3–353.0)
	**Ferritin < 100 ng/ml**	N (%)	68 (28.6%)
	**Ferritin < 500 ng/ml (%)**	N (%)	203 (85.3%)
	**TSAT** *,**	Median (IQR)	19.0% (14.0%– 28.0%)
	**TSAT < 20%** **	N (%)	81 (52.9%)
	TSAT < 30%**	N (%)	121 (79.1%)
	**Ferritin < 100 ng/ml and/or TSAT < 20%+**	N (%)	122 (68.5%)
	**Ferritin ≤500 ng/ml and TSAT ≤30% ++**	N (%)	114 (68.3%)
	Agreement**	Kappa (95%CI)£	.47 (.32 - .62)
**Imputed**	**TSAT** *,***	Median (IQR)	20.1% (13.6%– 28.8%)
	**TSAT < 20%** ***	N(%)	118(49.6%)
	TSAT < 30%***	N(%)	181(76.2%)
	Ferritin < 100 ng/ml and/or TSAT < 20%***	N(%)	146(61.5%)
	**Ferritin ≤500 ng/ml and TSAT ≤30%** ***	N(%)	167(70.2%)
	Agreement***	Kappa (95%CI)$	.47 (.32 - .62)

*: Distribution departed significantly from a normal distribution, thus the median and its Interquartile Range (IQR = Q1- Q3) were used.

**: Original data, 153 observations with non-missing values.

***: Average of 5 datasets with n = 238 each. Imputed values were derived by multiple imputation algorithms.

+: Due to the logical construction of the test, the denominator is 178.

++: Due to the logical construction of the test, the denominator is 167.

£: 95% Confidence interval by bootstrapping, based on 1000 bootstrap samples.

$: 95% Confidence interval using kappa ± 1.96 SE (no bootstrapping with multiple imputation).

### Patients’ characteristics by gender and CKD stage subgroups

Distribution of the patients’ characteristics by gender and CKD stage is summarized in Table 2. Of notice, mean hemoglobin level was comparable among women and men and in the different CKD stages. However, after adjusting for CKD stage (Table 3), female gender was significantly associated with hemoglobin level < 11 g/dL (Mantel-Haenzel adjusted OR 2.54 (95%CI 1.33–5.21)).

**Table 2:** Characteristics of the 238 patients by gender and CKD stage.

			CKD Stage 3		CKD Stage 4		CKD Stage 5
Variable							
	Sex	N	Median (IQR)	N	Median (IQR)	N	Median (IQR)
Age (years)*	M	21	76 (69–81)	36	75 (57–82)	34	67 (57–81)
	F	22	74 (60–80)	60	68 (58–78)	65	69 (62–76)
eGFR (ml/min)*	M	21	38 (35–47)	36	21 (17–25)	34	10 (8–12)
	F	22	35 (32–38)	60	20 (16–26)	65	10 (7–12)
**Hemoglobin (g/dl)£**	M	21	9.6 ± 1.2	36	9.5 ± 1.8	34	10.1 ± 1.4
	F	22	9.6 ± .9	60	9.6 ± 1.4	65	9.5 ± 1.4
Ferritin (ng/ml)*	M	21	230 (145–439)	36	178 (83–308)	34	166 (83–280)
	F	22	104 (77–203)	60	154 (77–351)	65	264 (89–429)
TSAT (%)*,**	M	9	22% (19% - 32%)	21	19% (15% - 24%)	25	18% (15% - 23%)
	F	14	16% (13% - 19%)	41	23% (14% - 30%)	43	19% (13% - 30%)

*: Distribution departed significantly from a normal distribution, thus the median and its Interquartile Range (IQR = Q1- Q3) were used.

**: Original data, 153 observations with non-missing values.

£: mean ± standard deviation, for Hemoglobin has a normal distribution.

**Table 3:** Hemoglobin and iron parameters of the 238 patients after adjusting for CKD stage.

			CKD Stage 3			CKD Stage 4			CKD Stage 5	
Variable										
	M/F	N	OR (95%CI)^+++^	M/F	N	OR (95%CI)^+++^	M/F	N	OR (95%CI)^+++^	MH OR ***
**Hb <10 g/dl**	21/22	43	1.59 (.46–7.33)	36/60	96	1.07 (.44–2.53)	34/65	34	2.14 (.95–5.21)	1.53 (.91–2.78)
**Hb <11 g/dl**	21/22	43	2.21 (.32–6.19)	36/60	96	2.20 (.75–6.31)	34/65	34	3.0 (1.03–8.78)	2.54 (1.33–5.21)
**Ferritin < 100 ng/ml**	21/22	43	2.94 (.77–16.9)	36/60	96	1.11 (.43–2.92)	34/65	34	.85 (.35–2.37)	1.20 (.65–2.28)
**Ferritin < 500 ng/ml**	21/22	43	6.56 (.72–15.3)	36/60	96	1.30 (.28–4.15)	34/65	34	.43 (.10–1.43)	1.10 (.46–2.29)
**TSAT < 20%** **	9/14	23	12 (1.80–79.0)	21/41	62	.71 (.24–2.24)	25/43	68	.70 (.25–2.05)	1.03 (.51–2.08) $
**TSAT < 30%** **	9/14	23	Non defined	21/41	62	.36 (.10–1.45)	25/43	68	.42 (.09–1.43)	.66 (.23–1.44) $
**Ferritin < 100 ng/ml and/or TSAT < 20%+**	11/17	28	9.0 (1.33–57.16)	25/49	74	.50 (.14–1.51)	27/49	76	.54 (.15–1.50)	.82 (.40–1.54) $
**Ferritin ≤500 ng/ml and TSAT ≤30%++**	10/15	25	21.0 (2.5–105)	24/45	69	.60 (.13–1.84)	25/48	73	.38 (.08–1.17)	.88 (.38–1.64) $

Unless stated otherwise, all 95%CI were derived by bootstrapping, based on 1000 bootstrap samples.

**: Original data, 153 observations with non-missing values.

***: Mantel-Haenszel Common Odds Ratio Estimate, adjusting for CKD stage, based on 1000 bootstrap samples.

+: Due to the logical construction of the test, the common denominator is 178.

++: Due to the logical construction of the test, the common denominator is 167.

+++: Reference category: male gender.

$: MH OR not reliable due to significant interaction.

Median ferritin levels, while decreasing with CKD stage in men, had the reverse pattern in women. After adjusting for CKD stage, ferritin levels < 100 ng/ml and < 500 ng/ml were not associated with female gender (respective Mantel-Haenzel adjusted OR 1.20 (95%CI .65–2.28) and 1.10 (95%CI.46 – 2.29)), although interaction cannot be completely excluded (p interaction = .081).

**Fig 1:**
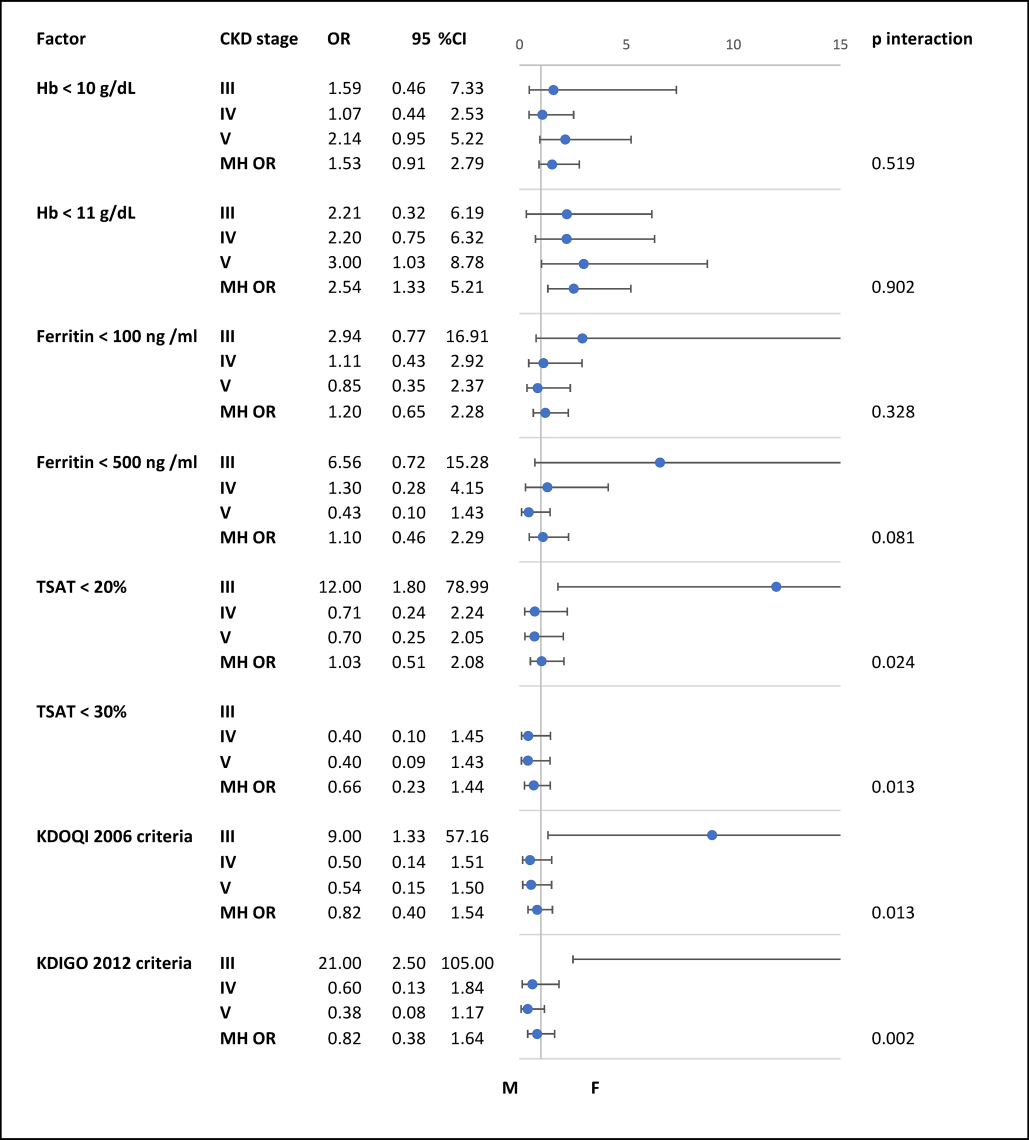
Comparison between men and women across different stages of chronic kidney disease. Abbreviations: Hb, hemoglobin; MH OR, Mantel-Haenszel Common Odds Ratio Estimate; KDOQI 2006 criteria, ferritin <100 ng/ml and/or TSAT<20%; KDIGO 2012 criteria, ferritin ≤500 ng/ml and TSAT ≤30%.

As compared to women in CKD stage 3, median TSAT was higher in men, while the pattern was reversed in CKD stage 4. The gender by CKD stage interaction was significant for TSAT < 20% and TSAT < 30%, and thus precluded deriving a usable Mantel-Haenzel adjusted OR. The latter situation translated in similar pattern and interpretation for the two definitions of iron deficiency, as shown in the forest plot of Fig 1.

### Main analysis

After adjustment on covariates (power transform of age and hemoglobin), INT of ferritin ranks tended to be statistically significant (p = 0.051, Table 4), mainly driven by the gender and CKD stage interaction.

**Table 4:** Analysis of covariance of inverse normal transform (INT) of ferritin rank (A) and TSAT rank (B).

**A: Ferritin**		
Source	Type I Sum of Squares	*p-*value
Corrected Model	13.363	.051
Intercept	8.856E-6	.998
Box Cox (Age)	1.311	.238
Hemoglobin	4.892	.023
Gender	.755	.370
CKD Stage	1.870	.370
Gender * CKD Stage	4.536	.091
Error	215.089	
Total	228.452	
Corrected Total	228.452	
**B: TSAT**		
Corrected Model	6.244	.479
Intercept	8.366E-7	.999
Box Cox (Age)	2.889	.083
Hemoglobin	3.477E-7	1.000
Gender	.216	.634
CKD Stage	.288	.860
Gender * CKD Stage	2.850	.227
Error	137.754	
Total	143.997	
Corrected Total	143.997	

Type 1 SS was used to adjust for unequal sample sizes in a non-experimental design.

A similar analysis of INT of TSAT ranks showed no significant association with gender or CKD stage.

### Sensitivity analysis

Additional analyses were performed on INT of TSAT using the 5 datasets generated by multiple imputation (S1 Table). Out of the 5 augmented datasets, 4 showed a significant overall effect, and the gender by CKD stage interaction seemed the most frequent significant association, potentially empowering the main ANCOVA of TSAT.

Furthermore, additional scenarios for the two definitions of iron deficiency were performed. In a first scenario, values were imputed using the 5 augmented datasets. In a second scenario, all missing values were set to ‘No’ (most conservative scenario). In a third scenario, all missing values were set to ‘Yes’ (the least conservative scenario). The results of these scenarios are shown in S2 Table in comparison with the original dataset.

According to these scenarios, iron requirement percentage based on KDOQI 2006 criteria would range between 51.3% and 76.5%, and between 47.9% and 77.7% based on KDIGO 2012 criteria, with a moderate agreement between the 2 sets of measures. However there was a trend towards more iron requirement while using KDIGO criteria. S3 Table depicts distribution of scenarios 1 to 3 according to gender and CKD stage.

## Discussion

This study highlights the high prevalence of iron deficiency in ND-CKD patients on ESA therapy. Remarkably it shows that the majority of female patients are iron deficient at the early stages of CKD but become more iron replenished in advanced stages. One plausible hypothesis is that women were not followed by a physician and were iron deficient when diagnosed with CKD then supplemented with iron during their follow-up. This finding is not surprising knowing that female gender is predictive for anemia and iron deficiency in the general population across most regions and age groups [2,3]. In CKD patients also, women usually display more iron deficiency than men when considering all CKD stages together [12,13]. Curiously, men in our study became more iron deficient when progressing from CKD stage 3 to CKD stages 4 and 5. It is very likely that men had no iron deficiency at the early CKD stages and were not or minimally substituted with iron while on ESA therapy. ESAs thus might have depleted their circulating iron progressively. This is the first time that a national study demonstrates a divergent evolution of iron deficiency between genders along CKD progression although at the limit of statistical significance.

Few researchers have previously demonstrated a high prevalence of iron deficiency based on ferritin<100 ng/ml and/or TSAT< 20% [12,13,14]. Minutolo et al studied CKD patients with eGFR<45 ml/min and found out more iron deficiency in women (68.6%) then men (53.8%) but they did not compare the gender difference across different CKD stages [12]. Cases-Amenos et al identified 36% of iron deficiency in ND-CKD patients on ESA therapy but they also did not draw a distinction between genders [14]. Fishbane et al were the only investigators to previously compare the prevalence of iron deficiency between men and women across different pre-dialysis CKD stages in a large American cohort [13]. However the only difference they found was a significant decrease in TSAT and increase in ferritin in women between early and advanced CKD stages. They could not attribute their finding to inflammation. Anyhow the decrease in their females’ TSAT is in contradiction with our findings where women increased simultaneously TSAT and ferritin. They found no difference in males across different CKD stages. Those discordant results between countries regarding the gender’s impact on iron deficiency need further analysis in a multinational investigation. It would be interesting to verify whether men in other countries and regions are more iron deficient than women in advanced CKD stages while on ESA therapy.

Furthermore, this work emphasizes the severity of anemia in CKD patients stages 3, 4 and 5. Anemia in CKD patients is recognized to be multifactorial. Anemia prevalence in CKD patients increases from 26% to 75% when the renal function decreases from > 60 ml/min to < 15 ml/min most probably because of EPO deficiency [3]. EPO deficiency is sometimes not only attributed to the kidney disease but also to another concomitant chronic disease [15]. Interestingly also, Mercadal et al have shown that anemia in CKD patients with eGFR>30 ml/min should be explained by other factors than EPO deficiency [16]. Treating patients with ESA while they suffer from other causes of anemia will lead to EPO resistance. Therefore, anemia in CKD patients when secondary to iron deficiency does not need ESA treatment unless it persists after iron replenishing [10]. All patients in our study were on ESA therapy but only 20% of them were above the KDIGO therapeutic target of 11 g/dl [10]. Unfortunately no data were available concerning the dose of ESA patients were taking when they applied for the treatment. Therefore we were not able to assess the chronic impact of ESA dose on anemia’s degree and on iron deficiency’s prevalence in our patients. Interestingly, Minutolo et al have shown equal iron deficiency prevalence in ND-CKD patients whether treated or not with ESAs despite the fact that iron supplementation was missed in a large number of their patients [12].

A remarkable finding of this study is the high prevalence of iron deficiency no matter of the definition used for ferritin and TSAT cutoffs. We identified however more patients with low TSAT than low ferritin but both iron indices progressed in a parallel way. Looking for iron deficiency in ND-CKD patients requires an assessment of iron indices, some being universally available and others difficult to perform practically [6]. Serum ferritin and TSAT are the most used markers [17]. It is believed that serum ferritin is a marker of iron stores and absolute iron deficiency and TSAT a measure of circulating available iron and reflects functional iron deficiency [18]. All international guidelines about iron deficiency recommend using serum ferritin for screening and one-half of them suggest adding TSAT [19]. Patients with heart failure for instance should be tested for both markers [20]. The renal guidelines usually combine both indices because CKD patients are believed to suffer from inflammation and thus functional iron deficiency. The UK-based National Institute for Health and Care Excellence (NICE) has proposed using red blood cell markers and if not available, a combination of TSAT <20% and ferritin level <100ng/mL as an alternative in CKD patients [11]. Some researchers would argue that serum ferritin, TSAT, serum soluble transferrin receptors and the serum soluble transferrin receptors-ferritin index are more accurate than classic red cell indices in the diagnosis of iron deficiency [1]. ERBP and NICE guidelines recommend treating ND-CKD patients with iron whenever ferritin <100 ng/ml and TSAT <20% [11]. The 2006 KDOQI guidelines recommend to maintain serum ferritin >100 ng/ml and TSAT >20% in ND-CKD patients on ESA therapy [4]. The 2012 KDIGO guidelines have set a higher target for ferritin in all CKD patients and recommend giving a trial of intravenous iron if TSAT is ≤30% and ferritin is ≤500 ng/ml [10]. TSAT seems to be more predictive of iron deficiency in CKD. An Italian study showed that TSAT <20% rather than ferritin <100 ng/ml predicted a decline in hemoglobin of 0.36 g/dl at 6 months [12]. In our series, adding TSAT to ferritin for the assessment of the iron status increased the prevalence of iron deficiency from 28% to 68.5%. Thus if we consider only TSAT as a marker of iron deficiency in our patients, this leads to an average of overall 53% prevalence of patients requiring iron therapy in all CKD stages.

The reasons behind the high prevalence of patients requiring iron therapy in ND-CKD patients are probably multifactorial. Clinical inertia in the management of iron deficiency in anemic CKD patients is a plausible cause. It was highlighted in a previous study where authors suggested that iron prescription was driven more by the hemoglobin level rather than the iron indices [12]. In a Spanish study on ND-CKD patients, 53% of the patients with iron deficiency were not prescribed iron supplements [14]. A study based on the NHANES data from 1988 to 2004 found low levels of iron indices in most patients with CKD [13]. A plausible explanation for all this iron deficiency worldwide is the primary general reluctance to prescribe iron at first place. In addition, patients not responding to oral therapy are not given intravenous iron. Some experts believe that intravenous iron may reduce or delay the use of ESAs in ND-CKD patients [21,22,23]. KDIGO suggested treating ND-CKD patients with intravenous iron if they were still iron deficient after three months of oral therapy [10]. Gotloib et al performed a bone marrow biopsy in CKD patients with Hb<12 g/dl and mean ferritin>200 ng/ml. They found iron deficiency in ~98% of the patients and intravenous iron corrected patients’ hemoglogin levels [24]. It is noteworthy that the FIND-CKD trial has shown that intravenous iron targeting low or high ferritin levels has no adverse events on the CKD progression [23].

This study has some strengths and limitations. It represents the Lebanese population because the MOPH covers the medication of half of the population with chronic diseases. Patients from all regions and socioeconomic status have access to this health coverage. The sample size is also representative of CKD patients in the country assuming that the prevalence of eGFR< 60 ml/min is ~8% [25] and the last adult population number estimated at 3.6 millions. It reflects also the practice of more than 80 nephrologists of the country. One limitation is the lack of information regarding the previous and current treatment of patients, specifically the dose of ESA and iron intake. Nevertheless a high percentage of patients had iron deficiency drawing attention to the undertreatment of this specific population and a high proportion of patients were screened for iron deficiency for the first time. Another caveat was due to missing TSAT values in one third of the patients. However multiple imputation algorithms and sensitivity analysis corroborated the hypothesis of these values missing at random and showed consistent estimates.

## Conclusion

Iron deficiency is highly prevalent in non-dialysis chronic kidney disease patients on ESA therapy. These findings reflect a lack in effective iron supplementation when managing anemia in pre-dialysis patients, especially in women at early stages and men at advanced stages. This clinical inertia regarding patients’ iron replenishment increases the use, the inefficacy and the costs of ESA therapy. Renal societies should spread awareness about iron deficiency screening with a combination of serum ferritin and TSAT in ND-CKD patients and third payers should enforce the application of international guidelines when approving ESA therapy in those patients.

## Supporting information

S1 Table ANCOVA of INT of TSAT performed on the original data and the 5 extra datasets generated by multiple imputation algorithm. (DOCX)

S2 Table Sensitivity analysis for KDOQI 2006 criteria and KDIGO 2012 criteria using different scenarios. (DOCX)

S3 Table Original data and scenarios 1 to 3 for transferrin saturation, KDOQI 2006 criteria, and KDIGO 2012 criteria, according to gender and CKD stage of the 238 patients. (DOCX)
