# Supplementary material for: Iron deficiency across chronic kidney disease stages: Is there a reverse gender pattern?
Source: PLoS One. 2018 Jan 22;13(1):e0191541. doi: 10.1371/journal.pone.0191541 (PMC5777643; doi:10.1371/journal.pone.0191541)
Supplement: S1 Table — (DOCX) [file pone.0191541.s001.docx]

**S1 Table. ANCOVA of INT of TSAT performed on the original data and the 5 extra datasets generated by multiple imputation algorithm.**

| **Tests of Between-Subjects Effects** | | | | | | | |
| --- | --- | --- | --- | --- | --- | --- | --- |
| Dependent Variable: Normal Score of TSAT using Van der Waerden's Formula | | | | | | | |
| Imputation Number | Source | Type I  Sum of Squares | df | Mean  Square | F | Sig. | Partial Eta Squared |
| Original data | Corrected Model | 6.244^a^ | 7 | .892 | .939 | .479 | .043 |
|  | Intercept | 8.366E-7 | 1 | 8.366E-7 | .000 | .999 | .000 |
|  | Box Cox Age | 2.889 | 1 | 2.889 | 3.041 | .083 | .021 |
|  | Hb | 3.477E-7 | 1 | 3.477E-7 | .000 | 1.000 | .000 |
|  | Gender | .216 | 1 | .216 | .228 | .634 | .002 |
|  | Stage | .288 | 2 | .144 | .152 | .860 | .002 |
|  | Gender * Stage | 2.850 | 2 | 1.425 | 1.500 | .227 | .020 |
|  | Error | 137.754 | 145 | .950 |  |  |  |
|  | Total | 143.997 | 153 |  |  |  |  |
|  | Corrected Total | 143.997 | 152 |  |  |  |  |
| 1 | Corrected Model | 10.561^c^ | 7 | 1.509 | 1.593 | .138 | .046 |
|  | Intercept | 4.632E-6 | 1 | 4.632E-6 | .000 | .998 | .000 |
|  | Box Cox Age | 7.526 | 1 | 7.526 | 7.946 | .005 | .033 |
|  | Hb | .046 | 1 | .046 | .049 | .826 | .000 |
|  | Gender | .579 | 1 | .579 | .612 | .435 | .003 |
|  | Stage | .347 | 2 | .173 | .183 | .833 | .002 |
|  | Gender * Stage | 2.062 | 2 | 1.031 | 1.089 | .338 | .009 |
|  | Error | 217.833 | 230 | .947 |  |  |  |
|  | Total | 228.394 | 238 |  |  |  |  |
|  | Corrected Total | 228.394 | 237 |  |  |  |  |
| 2 | Corrected Model | 17.135^d^ | 7 | 2.448 | 2.665 | .011 | .075 |
|  | Intercept | 2.786E-6 | 1 | 2.786E-6 | .000 | .999 | .000 |
|  | Box Cox Age | 7.714 | 1 | 7.714 | 8.399 | .004 | .035 |
|  | Hb | .023 | 1 | .023 | .025 | .875 | .000 |
|  | Gender | .326 | 1 | .326 | .355 | .552 | .002 |
|  | Stage | 5.117 | 2 | 2.558 | 2.785 | .064 | .024 |
|  | Gender * Stage | 3.955 | 2 | 1.977 | 2.153 | .118 | .018 |
|  | Error | 211.248 | 230 | .918 |  |  |  |
|  | Total | 228.383 | 238 |  |  |  |  |
|  | Corrected Total | 228.383 | 237 |  |  |  |  |
| 3 | Corrected Model | 14.611^e^ | 7 | 2.087 | 2.246 | .032 | .064 |
|  | Intercept | 4.685E-6 | 1 | 4.685E-6 | .000 | .998 | .000 |
|  | Box Cox Age | 11.018 | 1 | 11.018 | 11.854 | .001 | .049 |
|  | Hb | .037 | 1 | .037 | .039 | .843 | .000 |
|  | Gender | .147 | 1 | .147 | .158 | .691 | .001 |
|  | Stage | 2.411 | 2 | 1.206 | 1.297 | .275 | .011 |
|  | Gender * Stage | .998 | 2 | .499 | .537 | .585 | .005 |
|  | Error | 213.774 | 230 | .929 |  |  |  |
|  | Total | 228.385 | 238 |  |  |  |  |
|  | Corrected Total | 228.385 | 237 |  |  |  |  |
| 4 | Corrected Model | 23.264^f^ | 7 | 3.323 | 3.727 | .001 | .102 |
|  | Intercept | 2.390E-6 | 1 | 2.390E-6 | .000 | .999 | .000 |
|  | Box Cox Age | 7.062 | 1 | 7.062 | 7.920 | .005 | .033 |
|  | Hb | 2.504 | 1 | 2.504 | 2.808 | .095 | .012 |
|  | Gender | 4.398 | 1 | 4.398 | 4.932 | .027 | .021 |
|  | Stage | 1.553 | 2 | .777 | .871 | .420 | .008 |
|  | Gender * Stage | 7.746 | 2 | 3.873 | 4.343 | .014 | .036 |
|  | Error | 205.097 | 230 | .892 |  |  |  |
|  | Total | 228.361 | 238 |  |  |  |  |
|  | Corrected Total | 228.361 | 237 |  |  |  |  |
| 5 | Corrected Model | 23.754^g^ | 7 | 3.393 | 3.814 | .001 | .104 |
|  | Intercept | 2.612E-6 | 1 | 2.612E-6 | .000 | .999 | .000 |
|  | Box Cox Age | 6.280 | 1 | 6.280 | 7.059 | .008 | .030 |
|  | Hb | 2.023 | 1 | 2.023 | 2.273 | .133 | .010 |
|  | Gender | 3.759 | 1 | 3.759 | 4.225 | .041 | .018 |
|  | Stage | .148 | 2 | .074 | .083 | .920 | .001 |
|  | Gender * Stage | 11.544 | 2 | 5.772 | 6.488 | .002 | .053 |
|  | Error | 204.626 | 230 | .890 |  |  |  |
|  | Total | 228.380 | 238 |  |  |  |  |
|  | Corrected Total | 228.380 | 237 |  |  |  |  |
| a. R Squared = .043 (Adjusted R Squared = -.003) | | | | | | | |
| b. Computed using alpha = .05 | | | | | | | |
| c. R Squared = .046 (Adjusted R Squared = .017) | | | | | | | |
| d. R Squared = .075 (Adjusted R Squared = .047) | | | | | | | |
| e. R Squared = .064 (Adjusted R Squared = .035) | | | | | | | |
| f. R Squared = .102 (Adjusted R Squared = .075) | | | | | | | |
| g. R Squared = .104 (Adjusted R Squared = .077) | | | | | | | |
