# Supplementary material for: Iron deficiency across chronic kidney disease stages: Is there a reverse gender pattern?
Source: PLoS One. 2018 Jan 22;13(1):e0191541. doi: 10.1371/journal.pone.0191541 (PMC5777643; doi:10.1371/journal.pone.0191541)
Supplement: S2 Table — (DOCX) [file pone.0191541.s002.docx]

**S2 Table: Sensitivity analysis for KDOQI 2006 criteria and KDIGO 2012 criteria using different scenarios.**

| **Variable** | **Statistic** | **Original** | **Scenario 1** | **Scenario 2** | **Scenario 3** |
| --- | --- | --- | --- | --- | --- |
|  |  |  |  |  |  |
| **Sample size** | N(%) | 153(64%) | 238(100%) | 238(100%) | 238(100%) |
| **TSAT < 20 %** | N(%) | 81(52.9%) | 118(49.6%)* | - | - |
| **TSAT < 30 %** | N(%) | 121(79.1%) | 181(76.2%)* | - | - |
| **KDOQI 2006 criteria** | N(%) | 122(68.5%)+ | 146(61.5%)* | 122(51.3%) | 182(76.5%) |
| **KDIGO 2012criteria** | N(%) | 114(68.3%)++ | 167(70.2%)* | 114(47.9%) | 185(77.7%) |
| **Agreement** | Kappa (95%CI) | .47 (.32 - .62)£ | .47 (.32 - .62)$ | .50 (.39 - .61)£ | .42 (.27 - .55)£ |

Scenario 1: multiple imputation

Scenario 2: conservative imputation

Scenario 3: non-conservative imputation

*: Average of 5 datasets with n=238 each. Imputed values were derived by multiple imputation algorithms.

+: due to the logical construction of the test, the denominator is 178.

++: due to the logical construction of the test, the denominator is 167.

£: 95% Confidence interval by bootstrapping, based on 1000 bootstrap samples.

$: 95% Confidence interval using kappa ± 1.96 SE (no bootstrapping with multiple imputation).
