# Supplementary material for: Iron deficiency across chronic kidney disease stages: Is there a reverse gender pattern?
Source: PLoS One. 2018 Jan 22;13(1):e0191541. doi: 10.1371/journal.pone.0191541 (PMC5777643; doi:10.1371/journal.pone.0191541)
Supplement: S3 Table — (DOCX) [file pone.0191541.s003.docx]

**S3 Table: Original data and Scenarios 1 to 3 for transferrin saturation, KDOQI 2006 criteria, and KDIGO 2012 criteria, according to gender and CKD stage of the 238 patients.**

| **Variable** | **CKD Stage 3** |  | **CKD Stage 4** |  | **CKD Stage 5** |  | **Adjusted** |
| --- | --- | --- | --- | --- | --- | --- | --- |
|  | **M/F (N)** | **OR (95%CI) ^+++^** | **M/F (N)** | **OR (95%CI) ^+++^** | **M/F (N)** | **OR (95%CI) ^+++^** | **MH OR ***** |
| **Original dataset** |  |  |  |  |  |  |  |
| **TSAT < 20% **** | 9/14 (23) | 12.0(1.80 – 79.0) | 21/41 (62) | .71(.24 - 2.24) | 25/43 (68) | .70(.25 – 2.05) | 1.03(.51 – 2.08)$ |
| **TSAT < 30% **** | 9/14 (23) | undefined | 21/41 (62) | .36(.10 - 1.45) | 25/43 (68) | .42(.09 - 1.43) | .66 (.23 – 1.44)$ |
| **KDOQI 2006 criteria +** | 11/17(28) | 9.0(1.33 - 57.16) | 25/49(74) | .50(.14 - 1.51) | 27/49(76) | .54(.15 - 1.50) | .82 (.40 – 1.54)$ |
| **KDIGO 2012 criteria ++** | 10/15(25) | 21.0 (2.5 – 105) | 24/45(69) | .60 (.13 – 1.84) | 25/48(73) | .38 (.08 – 1.17) | .88 (.38 – 1.64)$ |
| **Scenario 1 (5 datasets)** |  |  |  |  |  |  |  |
| **TSAT < 20% £** | 21/22(43) | 3.56(.99 – 12.7) | 36/60(96) | .53(.23 - 1.23) | 34/65(99) | .86(.38 - 1.98) | .92 (.55 – 1.56)$ |
|  | 21/22(43) | 4.95(1.24 – 19.7) | 36/60(96) | .72(.31 – 1.68) | 34/65(99) | .86(.38 - 1.98) | 1.08 (.64 – 1.84)$ |
|  | 21/22(43) | 2.55(.68 – 9.54) | 36/60(96) | .51(.22 - 1.18) | 34/65(99) | .87(.38 - 1.99) | .84 (.50 – 1.43) |
|  | 21/22(43) | 14.4(3.29 – 63.1) | 36/60(96) | .96(.42 – 2.19) | 34/65(99) | .91(.39 – 2.08) | 1.45 (.87 – 2.43)$ |
|  | 21/22(43) | 14.5(3.30 – 63.3) | 36/60(96) | 1.02(.45 – 2.35) | 34/65(99) | .91(.39 – 2.08) | 1.50 (.89 – 2.51)$ |
|  |  |  |  |  |  |  |  |
| **TSAT < 30% £** | 21/22(43) | 7.50(1.38 – 40.7) | 36/60(96) | .44(.15 – 1.34) | 34/65(99) | .40(.10 - 1.59) | .89 (.48 – 1.66)$ |
|  | 21/22(43) | undefined | 36/60(96) | .71(.29 - 1.76) | 34/65(99) | .61(.21 - 1.71) | .94 (.50 – 1.75)$ |
|  | 21/22(43) | 3.13(.53 – 18.3) | 36/60(96) | .44(.15 - 1.34) | 34/65(99) | .66(.23 - 1.87) | .73 (.37 – 1.41) |
|  | 21/22(43) | undefined | 36/60(96) | .72(.26 – 1.99) | 34/65(99) | .75(.29 – 1.94) | 1.16 (.63 – 2.14)$ |
|  | 21/22(43) | 23.1(2.61 – 205) | 36/60(96) | 1.03(.42 – 2.52) | 34/65(99) | .49(.16 – 1.46) | 1.27 (.71 – 2.28)$ |
|  |  |  |  |  |  |  |  |
| **KDOQI** | 21/22(43) | 4.95(1.24- 19.7) | 36/60(96) | .58(.24 - 1.38) | 34/65(99) | .58(.24 - 1.42) | .87 (.50 – 1.49)$ |
| **2006 criteria £** | 21/22(43) | 6.97(1.57 – 30.9) | 36/60(96) | .73(.31 – 13.5) | 34/65(99) | .72(.30 - 1.72) | 1.05 (.62 – 1.80)$ |
|  | 21/22(43) | 3.38(.84 – 13.56) | 36/60(96) | .54(.23 - 1.29) | 34/65(99) | .62(.25 - 1.53) | .79 (.46 – 1.38)$ |
|  | 21/22(43) | 11.2(2.67 - 47.4) | 36/60(96) | .76(.31 - 1.84) | 34/65(99) | .72(.31 - 1.70) | 1.21 (.71 – 2.05)$ |
|  | 21/22(43) | 14.4(3.29 – 63.1) | 36/60(96) | .69(.30 - 1.61) | 34/65(99) | .72(.31 - 1.70) | 1.19 (.71 – 1.99)$ |
|  |  |  |  |  |  |  |  |
| **KDIGO** | 21/22(43) | 6.97(1.57 – 30.9) | 36/60(96) | .52(.19 - 1.40) | 34/65(99) | .60(.23 - 1.55) | .93 (.52 – 1.64)$ |
| **2012 criteria £** | 21/22(43) | 10.5(1.16 – 94.9) | 36/60(96) | .66(.27 – 1.63) | 34/65(99) | .65(.25 - 1.66) | .93 (.52 – 1.66)$ |
|  | 21/22(43) | 3.17(.69 – 14.5) | 36/60(96) | .56(.21 - 1.52) | 34/65(99) | .60(.23 - 1.55) | .79 (.43 – 1.44) |
|  | 21/22(43) | 19.1(2.16 – 169) | 36/60(96) | .56(.21 - 1.52) | 34/65(99) | .67(.27 - 1.63) | 1.02 (.58 – 1.80)$ |
|  | 21/22(43) | 16.3(2.97 – 88.9) | 36/60(96) | .93(.39 – 2.22) | 34/65(99) | .44(.17 - 1.17) | 1.14 (.66 – 1.95)$ |
| **Scenario 2** |  |  |  |  |  |  |  |
| **KDOQI 2006 criteria** | 21/22(43) | 6.86(1.94 – 35.9) | 36/60(96) | .90(0.40 – 2.07) | 34/65(99) | 0.60(0.23 - 1.39) | 1.10 (.64 – 1.83)$ |
| **KDIGO 2012 criteria** | 21/22(43) | 7.44(1.87 – 40.7) | 36/60(96) | .94(0.41 – 2.26) | 34/65(99) | .56(.22 - 1.40) | 1.09 (.66 – 1.83)$ |
| **Scenario 3** |  |  |  |  |  |  |  |
| **KDOQI 2006 criteria** | 21/22(43) | 4.00(.76 – 16.3) | 36/60(96) | .43(0.12 – 1.14) | 34/65(99) | .61(0.16 - 1.67) | .72 (.36 – 1.30) |
| **KDIGO 2012 criteria** | 21/22(43) | 8.40(1.06 – 20.6) | 36/60(96) | .55(.13 – 1.55) | 34/65(99) | .42(.10 - 1.20) | .73 (.38 – 1.40) $ |
|  |  |  |  |  |  |  |  |

Unless stated otherwise, all 95%CI were derived by bootstrapping, based on 1000 bootstrap samples.

**: Original data, 153 observations with non-missing values.

+: due to the logical construction of the test, the common denominator is 178.

++: due to the logical construction of the test, the common denominator is 167.

+++: reference category: male gender.

$: MH OR not reliable due to significant interaction

£: No bootsrapping
